# Early Predictive Value of NT-proBNP Combined With Echocardiography in Anthracyclines Induced Cardiotoxicity

**DOI:** 10.3389/fsurg.2022.898172

**Published:** 2022-07-01

**Authors:** Yingjun Dong, Qiong Wu, Changqing Hu

**Affiliations:** ^1^ Shanxi Province Cancer Hospital/Shanxi Hospital Affiliated to Cancer Hospital, Chinese Academy of Medical Sciences/Cancer Hospital Affiliated to Shanxi Medical University, Taiyuan, China; ^2^ Department of Cardiology, Shanxi Provincial People’s Hospital, Taiyuan, China

**Keywords:** cardiac toxicity, anthracyclines, N-terminal B-type natriuretic peptide, cardiac ultrasound, early predictive value

## Abstract

**Objective:**

Determine the predictive value of N-terminal pro-B-type natriuretic peptide (NT-proBNP) combined with echocardiography in the diagnosis of anthracyclines-induced chronic cardiotoxicity.

**Methods:**

A total of 80 female breast cancer patients from January 2019 to October 2021 were included in our hospital. Twenty-six patients with cardiotoxicity were divided into the cardiac impairment group, and the 54 patients without cardiotoxicity were classified into the normal control group. NT-proBNP levels and cardiac echocardiography were measured before the start of the chemotherapy cycle, in cycle 3 of the chemotherapy, and after the chemotherapy cycle in all patients.

**Results:**

After three cycles of chemotherapy and chemotherapy, the levels of NT-proBNP in patients of the two groups were significantly higher than those before chemotherapy (*P *< 0.05). The levels of NT-proBNP in the cardiac injury group after three cycles of chemotherapy and chemotherapy were higher than those in the normal control group at the same time point (*P *< 0.05). The LVEF of patients in the cardiac impairment group after chemotherapy was lower than that before chemotherapy, and the LVEF after chemotherapy was lower than that in the normal control group (*P *< 0.05). NT-proBNP had a negative correlation with LVEF (*r *= −0.549, *P *< 0.001). The AUC of NT-proBNP in combination with LVEF for predicting cardiotoxicity in our patient was 0.898(95%CI:0.829–0.966).

**Conclusion:**

NT-proBNP combined with echocardiography has clinical significance in the detection of anthracyclines-induced cardiotoxicity, and it can detect early myocardial injury induced by anthracyclines, with early prediction value. It is important to protect heart function and judge prognosis.

## Introduction

Breast cancer is the most common malignant tumor among women in the world, and anthracycline, doxorubicin and other drugs are the most commonly used chemotherapy drugs after breast cancer surgical (1). anthracyclines drugs are widely used in the treatment of hematological malignancies and solid tumors, effectively reducing the mortality rate of malignant tumors and prolonging the overall survival period (2). anthracyclines, as a classic first-line chemotherapy drug, has dose-related cardiotoxicity. Most patients treated with anthracycline showed early or late cardiac insufficiency, and the main manifestations of ECG were arrhythmia, ST segment changes or conduction block (3, 4). At the same time, due to the limitation of anthracycline’s cardiotoxicity, its cumulative dose in patients is greatly limited, and many patients stop chemotherapy because of cardiac dysfunction during chemotherapy. This directly affects the quality of life and the incidence of long-term cardiac death of patients receiving chemotherapy (5, 6). Therefore, early monitoring and prevention of anthracyclines-induced cardiotoxicity events are particularly important.

A nearly perfect diagnostic tool for evaluating anthracycline-associated cardiotoxicity needs to meet the characteristics of non-invasiveness, high sensitivity, and low cost. Left ventricular ejection fraction (LVEF) is a commonly used index to monitor ventricular systolic and diastolic function, which can be assessed by echocardiography. At the same time, echocardiography can intuitively show the anatomical structure and hemodynamic changes of the heart, and detect the systolic and diastolic functions of the heart (7). Many studies have shown that heart-specific biomarkers such as N-terminal pro-B-type natriuretic peptide (NT-proBNP) may be valuable in identifying high-risk patients (8–10). The etection of NT-proBNP is convenient, quick and cheap. The purpose of this study is to study and effectively analyze the cardiotoxicity of anthracycline chemotherapy in breast cancer patients by using NT-proBNP and cardiac ultrasound monitoring, and to clarify the predictive value of non-invasive evaluation tools in preventing cardiotoxicity, and to guide clinical practice. The report is shown below.

## Data and Methods

### General Information

A total of 80 female breast cancer patients from January 2019 to October 2021 were included in our hospital. All patients ranged in age from 30–75 years and weighed 44–80 kg, including 23 patients with hypertension, 19 patients with diabetes and 22 patients without basic disease. Among these 80 patients, 26 patients with cardiotoxicity were classified as the cardiac impairment group, and 54 patients without cardiotoxicity were classified as the normal control group. Diagnostic criteria for cardiotoxicity events are a decrease in LVEF ≥10% measured by radionuclide myocardial perfusion imaging during or at the end of chemotherapy, or an absolute value of LVEF measured by radionuclide after chemotherapy ≤50%. This study was approved by the hospital ethics committee and the patients’ informed consent was obtained.

### Inclusion Criteria

(1) complete 6 cycles of chemotherapy; (2) those who have not received chemotherapy with other drugs in the past; (3) normal function of liver and kidney; (4) anthracycline antineoplastic drugs are mainly used in chemotherapy; (5) no obvious abnormality was found in echocardiography before chemotherapy.

### Exclusions

(1) previous coronary heart disease, valvular heart disease, congenital heart disease, malignant arrhythmia, etc.; (2) before entering the group, receiving chemotherapy or local radiotherapy; (3) withdrawal of drugs due to poor curative effect or intolerance of anthracycline drugs; (4) combined with other cardiotoxic drugs.

### Research Methods

Patients were treated with adjuvant chemotherapy with 6 cycles of pirarubicin + cyclophosphamide + fluorouracil or 6 cycles of epirubicin + docetaxel. NT-proBNP levels and cardiac echocardiography were measured before the start of the chemotherapy cycle, in cycle 3 of the chemotherapy, and after the chemotherapy cycle in all cases.

Five milliliters of venous blood was drawn from patients in the morning under fasting conditions, centrifuged at 3500 r/min for 10 min to separate plasma, and NT-proBNP was measured using a chemiluminescent immunoassay.

Echocardiography: Philips IE33 color Doppler ultrasound diagnostic apparatus, s5-1 ultrasound probe with probe frequency of 2.5–4.0 MHz. The patient was placed in the left inclined 30° decubitus position and all sections of the heart were routinely explored. The cardiac morphology, atrioventricular size, valvular activity and left ventricular contraction were observed by two-dimensional echocardiography. The end-diastolic diameter (LVDd) and end-systolic diameter (LVDs) were measured. The left ventricular LVEF was measured by two-plane modified Simpson’s method using a four-chamber cusp incision.

### Statistical Methods

SPSS22.0 software was used for processing. The experimental data were in accordance with the normal distribution, and the measurement data were expressed as mean standard deviation ($\bar{x}\pm s$). The t test was used for pairwise comparison. The count data were expressed as (%) and the comparison was performed using *χ^2^* test. Pearson’s method was used to analyze the correlation between NT-proBNP and LVEF. The ROC curve was used to evaluate the predictive value of NT-proBNP and LVEF for cardiac toxicity events. The test level was α = 0.05, and *P *< 0.05 indicated that the difference was statistically significant.

## Results

### Comparison of General Data Between the Two Groups

There was no significant difference in general information such as age, weight, basic disease and tumor type between the two groups (*P *> 0.05). As shown in Table 1.

**Table 1:** Comparison of general data between the two groups.

Group	*N*	Age (years)	Body weight (kg)	Basic disease (case)		Tumor type (case)	
				hypertension	diabetes	Invasive ductal carcinoma	Invasive lobular carcinoma
Cardiac impairment group	26	52.07 ± 5.96	59.86 ± 7.52	8	7	16	10
Normal control group	54	53.84 ± 6.27	60.28 ± 7.39	15	12	39	15
*t*/*χ*^2^ value		1.201	0.237	0.019		0.932	
*P* value		0.233	0.814	0.890		0.334	

### Changes of NT-proBNP Level in Two Groups

**Figure 1:**
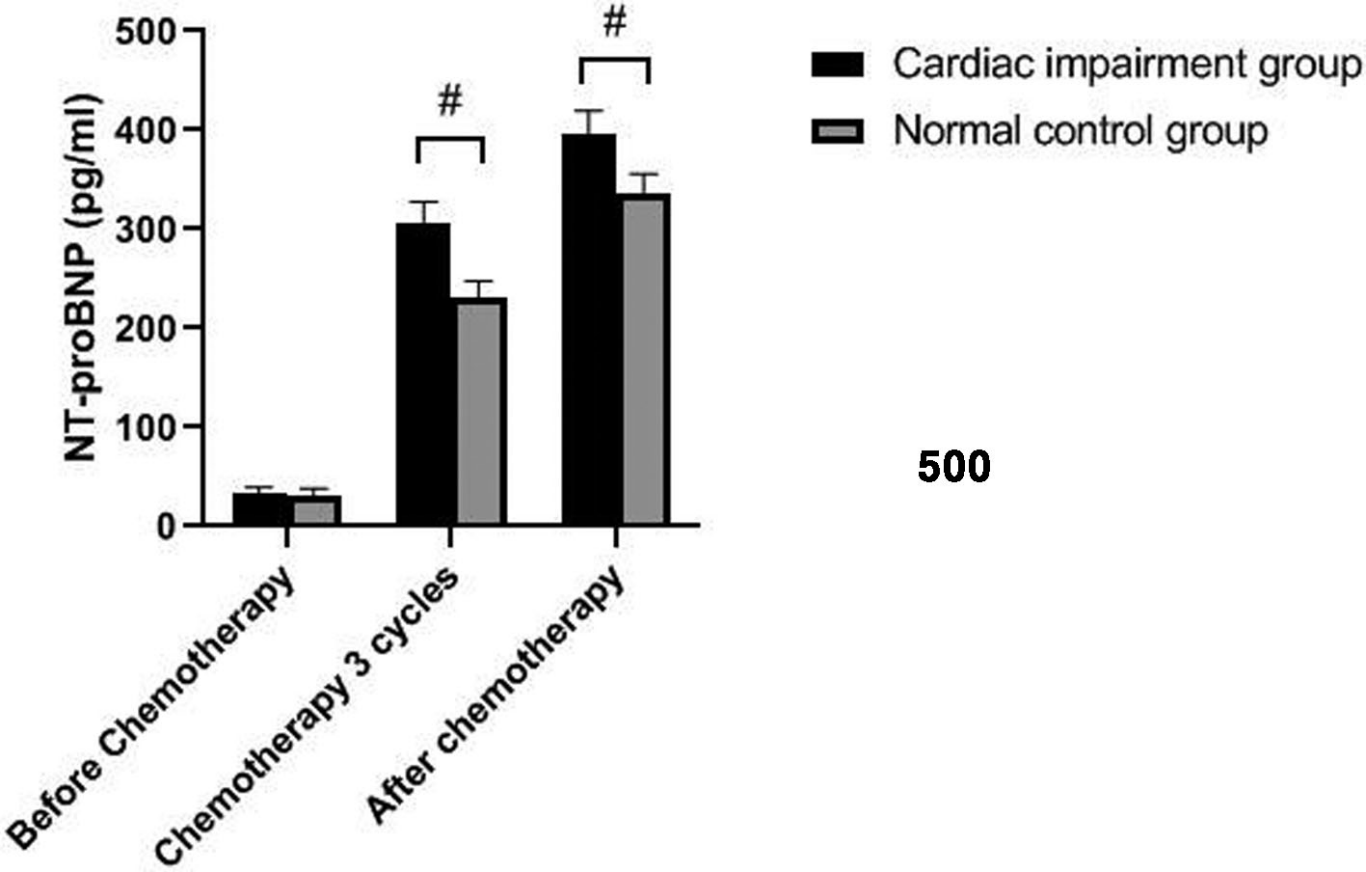
Comparison of NT-proBNP levels in two groups of patients at different time points. Note: Compared with the normal control group, ^#^*P *< 0.05.

**Figure 2:**
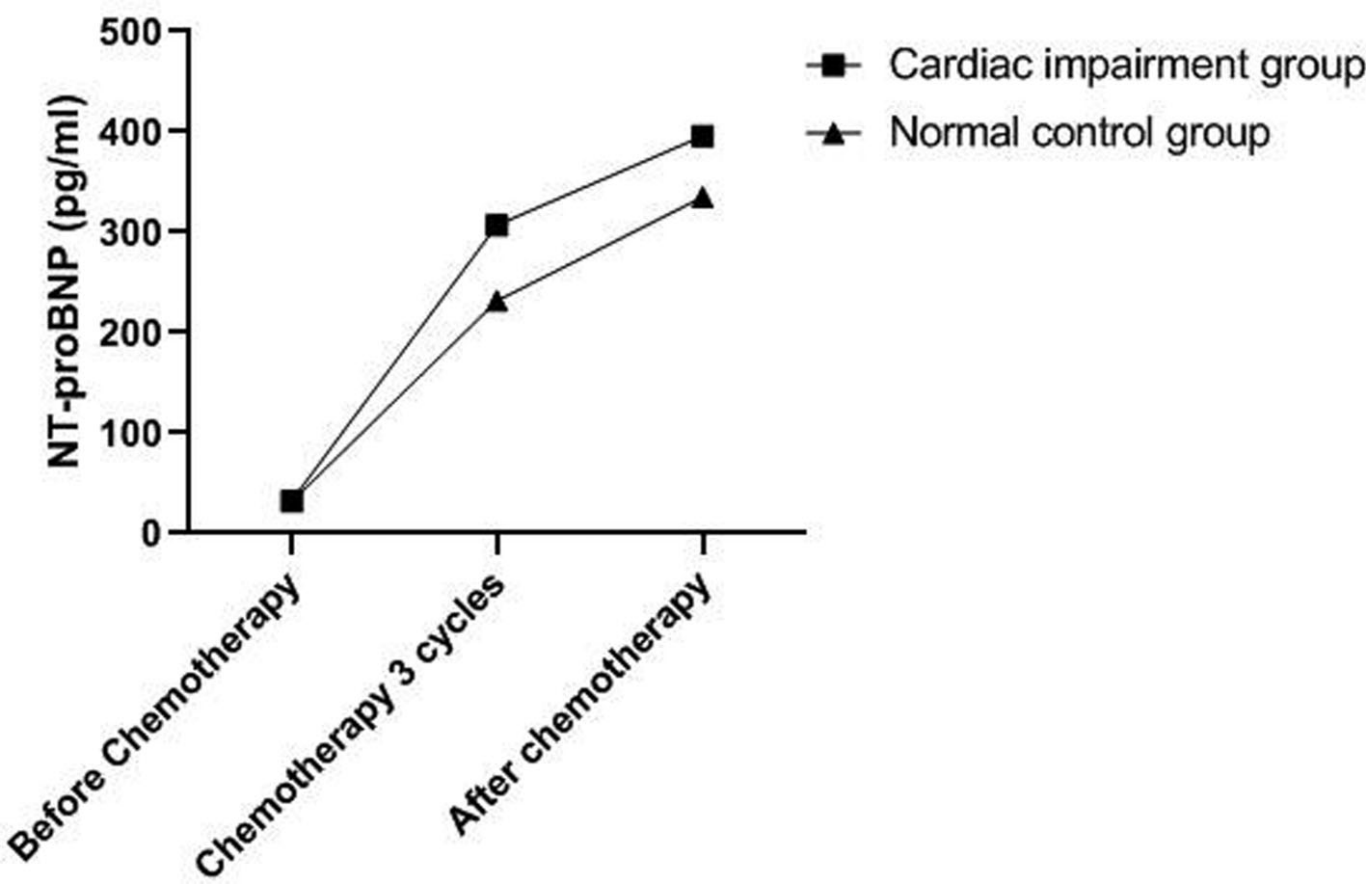
Change trend of NT-proBNP level in patients of two groups.

The levels of NT-proBNP in patients of the two groups after three cycles of chemotherapy and chemotherapy were significantly higher than those before chemotherapy (*P *< 0.05). The levels of NT-proBNP in the cardiac impairment group after three cycles of chemotherapy and chemotherapy were higher than those in the normal control group at the same time point (*P *< 0.05). As shown in Figures 1, 2.

### Changes of Echocardiography in Patients of Two Groups

**Figure 3:**
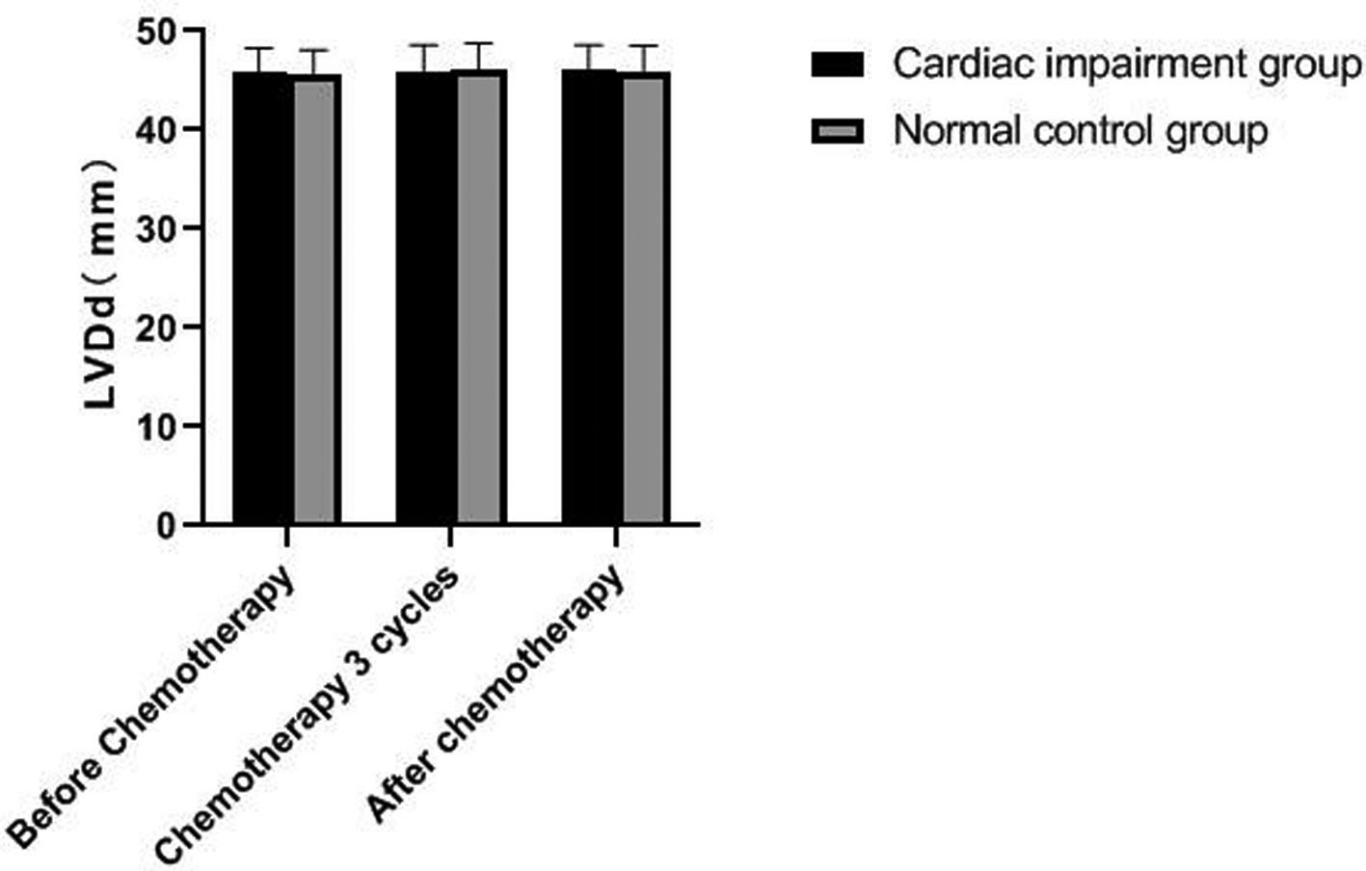
Comparison of LVDd values between the two groups at different time points.

**Figure 4:**
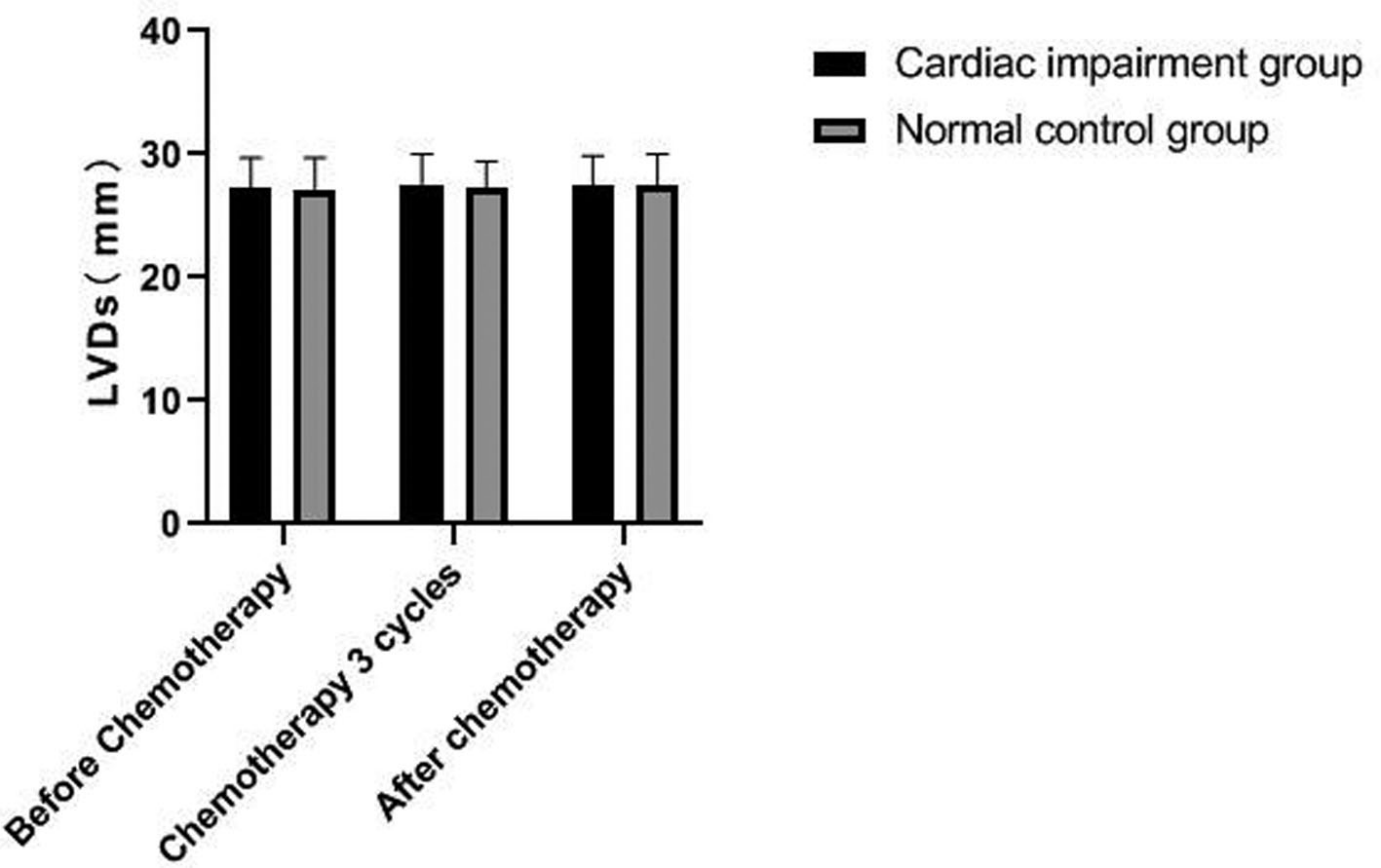
Comparison of LVDs values between the two groups at different time points.

**Figure 5:**
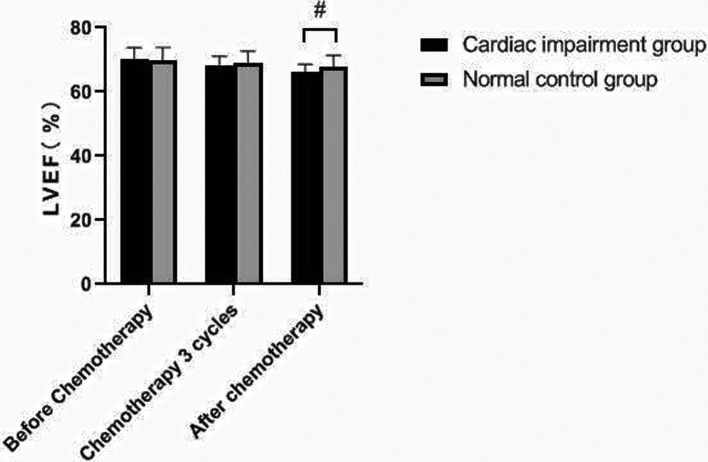
Comparison of LVEF values between the two groups at different time points. Note: Compared with the normal control group, ^#^*P *< 0.05.

**Figure 6:**
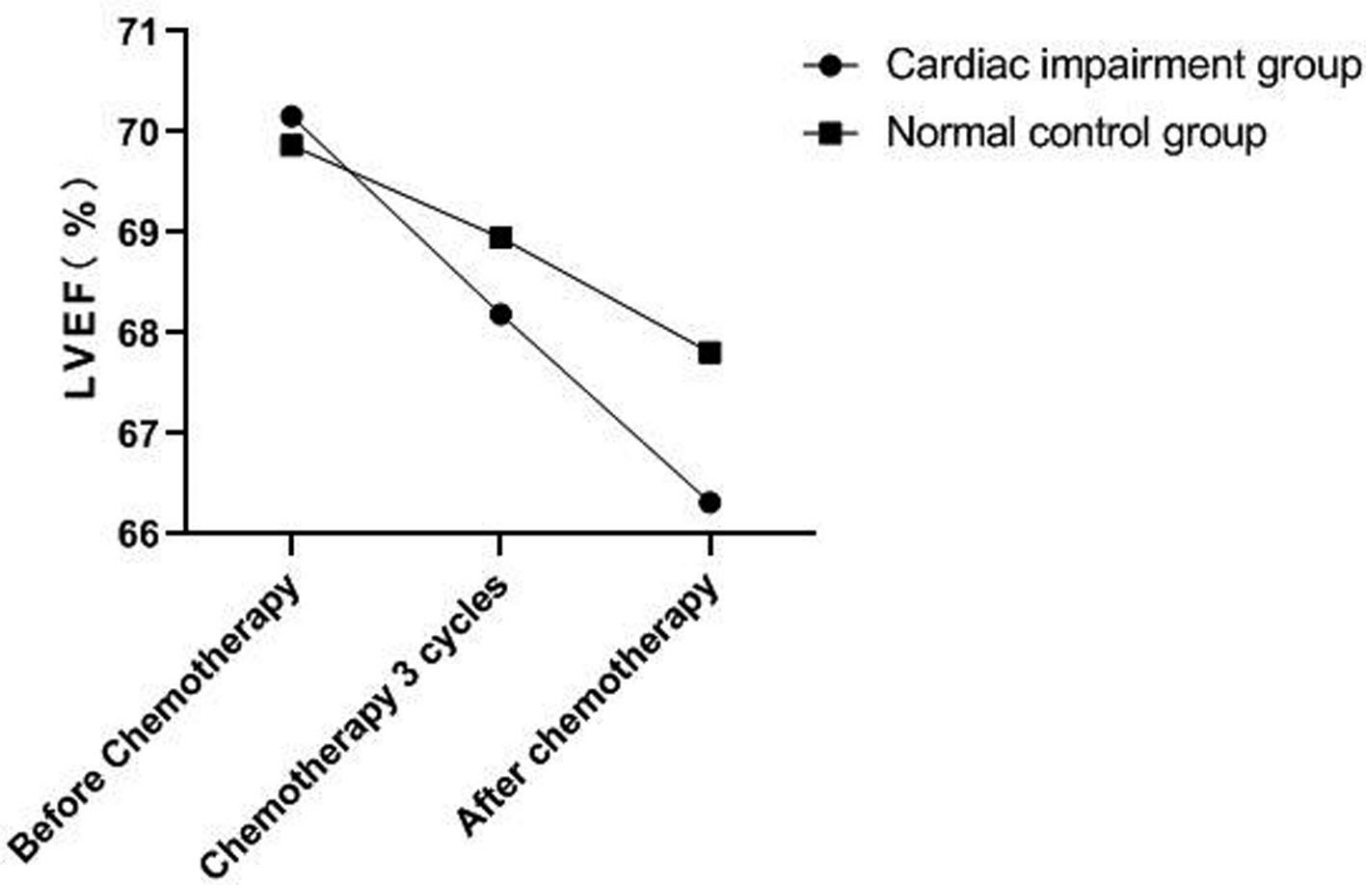
LVEF change trend of patients in two groups.

There was no significant change in LVDd and LVDs values before and during the 3rd cycle of chemotherapy and after chemotherapy between the two groups, and there was no difference between the two groups (*P *> 0.05). The LVEF of patients in the cardiac impairment group after chemotherapy was lower than that before chemotherapy, and the LVEF after chemotherapy was lower than that in the normal control group, and the differences were statistically significant (*P *< 0.05). As shown in Figures 3–6.

### Correlation Between NT-proBNP and LVEF

**Figure 7:**
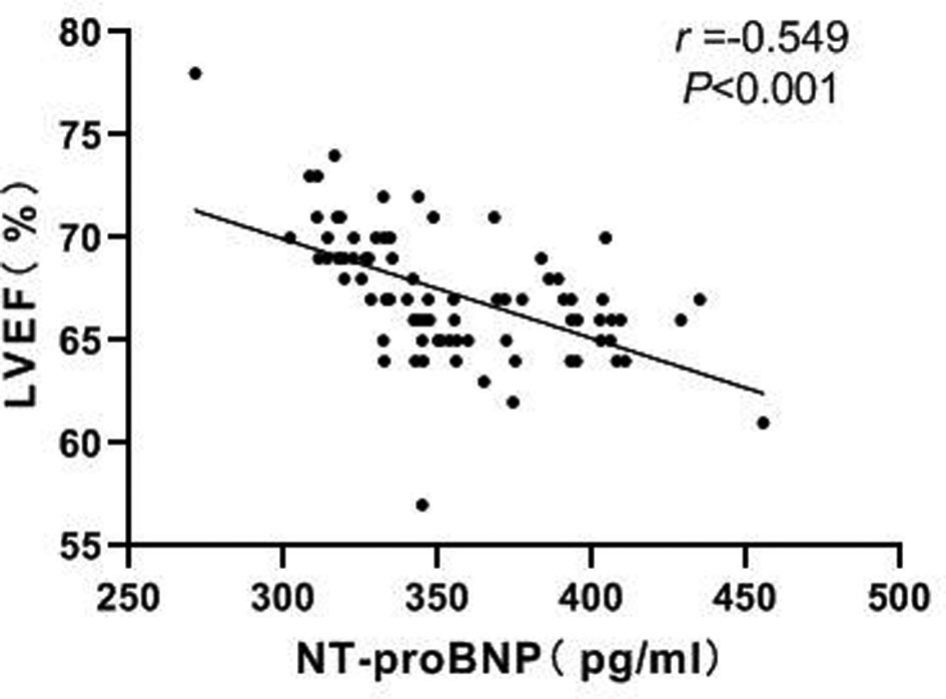
Correlation between NT-proBNP and LVEF.

Pearson correlation analysis showed a negative correlation between NT-proBNP and LVEF (*r *= −0.549, *P *< 0.001). As shown in Figure 7.

### Prediction Value of NT-pro BNP and Echocardiography Monitoring on Cardiac Toxicity in Patients

**Figure 8:**
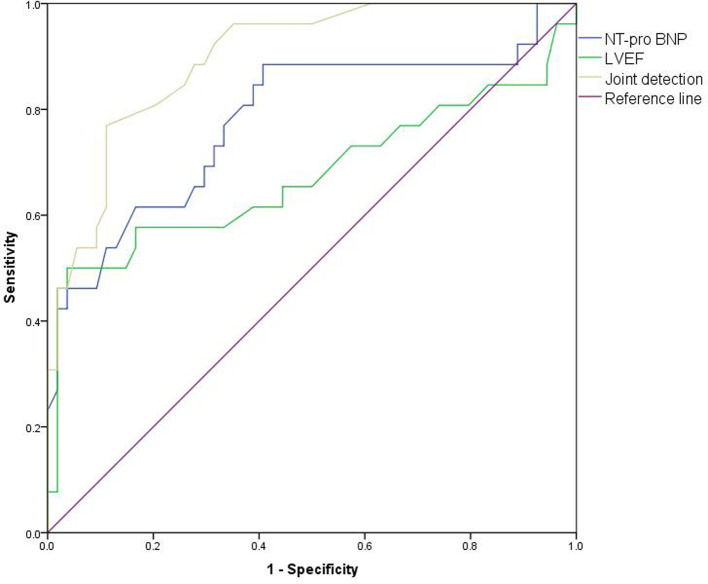
ROC curve of predictive value of NT-proBNP and echocardiography monitoring on cardiotoxicity of patients.

The AUC of NT-proBNP in combination with LVEF for predicting cardiotoxicity in our patient was 0.898 (95%CI:0.829–0.966), higher than the AUC of NT-proBNP of 0.780 (95% CI: 0.660–0.901) and LVEF of 0.675 (95% CI: 0.526–0.823). As shown in Figure 8.

## Discussion

Anthracyclines is a kind of cytotoxic drug, which has been widely used in anti-tumor therapy. It can significantly improve the survival rate of patients with breast cancer and other malignant tumors, and has important positive value in prolonging their survival (11). However, in cancer patients treated with anthracyclines chemotherapy drugs, due to the cardiotoxicity of the drug metabolite 1,3-dihydro derivatives, and the influence of susceptible factors such as oxygen free radicals, calcium overload and mitochondrial damage, the excessive accumulation can lead to serious cardiotoxicity, especially in patients with underlying heart diseases in the past (12). At present, more and more evidences (citations) have supported the cardiotoxicity of anthracyclines and their clinical relevance (13–15). The study found that in patients after treatment with anthracyclines, drug-induced congestive heart failure and cardiomyopathy mostly occurred within 0–231d after treatment, and the severity depended on the cumulative dose (16).

The cardiotoxicity caused by anthracyclines is generally divided into acute, chronic and delayed, which represent different pathological processes of myocardial injury. Because the early clinical manifestations of cardiotoxicity are not obvious, once it is diagnosed, obvious myocardial lesions, with poor prognosis and high mortality (17–19). Therefore, during chemotherapy, it is necessary to monitor the cardiac status and toxicity of anthracyclines, find out and intervene in time, and improve the survival rate of patients after chemotherapy.

B-type natriuretic peptide (BNP) is mainly synthesized and secreted by ventricles and contains 32 amino acids, which is a kind of polypeptide cardiac neurohormone. Its release is closely related to ventricular volume expansion and ventricular pressure load, and it can reflect changes of left ventricular function. It is a good biochemical index for clinical diagnosis of heart failure, and reflects the grading of heart function (20). NT-proBNP and BNP were derived from the same precursor. The concentration of BNP can not only reflect the severity of cardiac insufficiency, but also its inactive product NT-proBNP has the same effect. Compared with BNP, NT-Pro BNP has a longer half-life and is more stable, with higher sensitivity and specificity, thus making it easier to achieve the standardization of detection (21–23). This study showed that the levels of NT-proBNP in the two groups were significantly higher after three cycles of chemotherapy and after chemotherapy, and the level of NT-proBNP in the cardiac damage group were significantly higher than those in the normal control group. These results suggest that NT-proBNP played an important role in the early detection of anthracycline cardiotoxicity. Romano et al. (24) studied the predictive value of serum NT-proBNP on long-term cardiotoxicity in low-dose chemotherapy regimens for breast cancer. Through the follow-up observations of 3 months, 6 months and 12 months, the left wentricular function damage in the group with continuously increased of serum NT-proBNP was significantly different from that in the group without increased serum NT-proBNP.

At present, left ventricular function can be evaluated by various non-invasive imaging techniques, including echocardiography. Echocardiography has the advantages of price advantage, non-invasive, simple operation and good repeatability, etc. It can not only visually display the shape, structure and movement of the heart, but also quantitatively display the systolic and diastolic function of the heart. It has been widely used in clinical and research for the detection of cardiac function and early assessment of myocardial damage (25, 26). There are many indexes for evaluating cardiac function. Clinically, LVEF is often used to estimate the systolic function of the left wentricle. The decline of LVEF represents irreversible damage, which is of great significance in the evaluation of left ventricular function (27). The results of this study show that the LVEF value of the cardiac impairment group after chemotherapy was lower than that of the normal control group, but the measurement method of LVEF by two-dimensional echocardiography is easy to change, and the confidence interval is 11%. In this study, LVEF decreased by 3.84% after chemotherapy, Considering the individual differences of patients, it cannot be used as a specific indicator of left ventricular systolic function decline in breast cancer patients after anthracyclines therapy.

LVEF is not very sensitive to the early detection of subclinical cardiac injury, and its parameter changes depend on myocardial contractility, preload and afterload, so it is less specific. Further analysis is needed in combination with other clinical indicators (28). Analysis of the value of NT-proBNP and LVEF in predicting cardiotoxic events in patients revealed that AUC 0.898(0.829–0.966) of NT-proBNP combined with LVEF was significantly higher than AUC 0.780 (0.660–0.901) of NT-proBNP and AUC 0.675 (0.526–0.823) of LVEF. The results confirmed the value of NT-proBNP combined with echocardiography in the early diagnosis of cardiac toxicity.

In summary, NT-proBNP combined with echocardiography monitoring has clinical significance in detecting anthracyclines-induced cardiotoxicity, and it can detect myocardial injury caused by anthracyclines at an early stage, which has the value of early prediction and is of great significance in protecting cardiac function and judging prognosis.

## Data Availability

The original contributions presented in the study are included in the article/Supplementary Material, further inquiries can be directed to the corresponding author/s.
